# Seropositivity for pathogens associated with chronic infections is a risk factor for all-cause mortality in the elderly: findings from the Memory and Morbidity in Augsburg Elderly (MEMO) Study

**DOI:** 10.1007/s11357-020-00216-x

**Published:** 2020-07-09

**Authors:** Marius Zeeb, Tobias Kerrinnes, Luka Cicin-Sain, Carlos A. Guzman, Wolfram Puppe, Thomas F. Schulz, Annette Peters, Klaus Berger, Stefanie Castell, André Karch

**Affiliations:** 1 Institute for Medical Information Science, Biometry and Epidemiology, Ludwig Maximilians University, Munich, Germany; 2 Pettenkofer School of Public Health, Munich, Germany; 3 Department for Epidemiology, Helmholtz Centre for Infection Research, Brunswick, Germany; 4 Department of Vaccinology and Applied Microbiology, Helmholtz Centre for Infection Research, Braunschweig, Germany; 5 Cluster of Excellence RESIST (EXC 2155), Hannover Medical School (MHH), Hannover, Germany; 6 Centre for Individualised Infection Medicine (CIIM), a joint venture of HZI and MHH, Hannover, Germany; 7 German Centre for Infection Research (DZIF), Hannover-Braunschweig site, Braunschweig, Germany; 8 Institute for Virology, Hannover Medical School (MHH), Hannover, Germany; 9 German Research Center for Environmental Health, Munich, Germany; 10 Institute of Epidemiology and Social Medicine, University of Münster, Münster, Germany

**Keywords:** All-cause mortality, Elderly, *Helicobacter pylori*, CMV, *Borrelia burgdorferi* sensu *lato*

## Abstract

Immunostimulation by chronic infection has been linked to an increased risk for different non-communicable diseases, which in turn are leading causes of death in high- and middle-income countries. Thus, we investigated if a positive serostatus for pathogens responsible for common chronic infections is individually or synergistically related to reduced overall survival in community dwelling elderly. We used data of 365 individuals from the German MEMO (Memory and Morbidity in Augsburg Elderly) cohort study with a median age of 73 years at baseline and a median follow-up of 14 years. We examined the effect of a positive serostatus at baseline for selected pathogens associated with chronic infections (*Helicobacter pylori*, *Borrelia burgdorferi* sensu *lato*, *Toxoplasma gondii*, cytomegalovirus, Epstein-Barr virus, herpes simplex virus 1/2, and human herpesvirus 6) on all-cause mortality with multivariable parametric survival models. We found a reduced survival time in individuals with a positive serostatus for *Helicobacter pylori* (accelerated failure time (AFT) − 15.92, 95% CI − 29.96; − 1.88), cytomegalovirus (AFT − 22.81, 95% CI − 36.41; − 9.22) and *Borrelia burgdorferi* sensu *lato* (AFT − 25.25, 95% CI − 43.40; − 7.10), after adjusting for potential confounders. The number of infectious agents an individual was seropositive for had a linear effect on all-cause mortality (AFT per additional infection − 12.42 95% CI − 18.55; − 6.30). Our results suggest an effect of seropositivity for *Helicobacter pylori*, cytomegalovirus, and *Borrelia burgdorferi* sensu *lato* on all-cause mortality in older community dwelling individuals. Further research with larger cohorts and additional biomarkers is required, to assess mediators and molecular pathways of this effect.

**Electronic supplementary material:**

The online version of this article (10.1007/s11357-020-00216-x) contains supplementary material, which is available to authorized users.

## Introduction

Chronic infections caused by bacteria and viruses like, e.g., *Mycobacterium tuberculosis* or the Human Immunodeficiency Virus, play an important role for human morbidity and mortality; both pathogens remain among the top causes of years of live lost worldwide (GBD 2018). While in these two infections pathogenic structures as well as the targeted immune response to the pathogen affect the health of the host directly, there is increasing evidence that chronic infections can also indirectly trigger non-communicable diseases (NCD) in the host, especially in the elderly (Ogoina and Onyemelukwe 2009). A suggested pathway of how chronic infections lead to NCDs is long-term immunostimulation (De Martinis et al. 2005; Mackowiak 1984; Speck and Ganem 2010) although the exact causal mechanism from infection to NCD is often difficult to understand, especially if more than one infectious agent is involved (O'Connor et al. 2006).

Several viruses, bacteria, and protozoa have been linked to long-term immunostimulation. Cytomegalovirus (CMV), a herpes virus with seroprevalence levels between 31.8 and 77.6% in Western Europe (Lachmann et al. 2018), has been shown to induce chronic antigenic stress (Pawelec and Gouttefangeas 2006), which accelerates immunosenescence (Aw et al. 2007; Solana et al. 2012). Other herpes viruses, like Epstein-Barr-Virus (EBV), the first identified human oncovirus (Epstein et al. 1964), herpes simplex virus 1/2 (HSV-1/2) (Bergstrom et al. 1990; Piacentini et al. 2014) and human herpesvirus 6 (HHV-6) (Eliassen et al. 2018) have been linked to the development of cancer and neurological diseases via immunostimulation. Bacterial pathogens, like *Helicobacter pylori* (*H. pylori*), the causative agent of gastric cancer and ulcer, and *Borrelia burgdorferi* sensu *lato* (*B. burgdorferi s.l.*) have been identified as initiators of changes in the immune system (Bartchewsky Jr. et al. 2009; Bransfield 2018; Sharma et al. 1995) with long-term NCD sequelae. The protozoan *Toxoplasma gondii* (*T. gondii*) causes a strong pro-inflammatory response which affects the brain; the consequences are still not fully understood (Lang et al. 2018; Miller et al. 2009).

More and more evidence emerges suggesting that seropositivity to pathogens responsible for chronic infections like CMV might be linked to all-cause mortality (Simanek et al. 2011). However, the current literature lacks long-term cohort studies, which examine not only the effect of one but also of a combination of several chronic infections on all-cause mortality in the elderly. The aim of our study was to investigate the effect of the serostatus of seven pathogens associated with chronic infections (CMV, HHV-6, EBV, HSV-1/2, *H. pylori*, *T. gondii*, and *B. burgdorferi s.l.*) on all-cause mortality using data of 365 older individuals with a median follow-up of 14 years from the Memory and Morbidity in Augsburg Elderly (MEMO) Study. The seven pathogens were selected because all of them are closely associated with chronic infections and are sufficiently prevalent in the source population to be assessed in the context of this study.

## Methods

### Study population

We used data from the MEMO study (*n* = 385), a German cohort of individuals between 65 and 83 years of age at baseline (Braune and Berger 2005). MEMO is a subcohort of the MONICA S2 cohort (WHO 1988), focusing specifically on diseases of the elderly. Study participants were phenotyped in Augsburg, Germany in 1997/1998 (start of follow-up) including collection of blood. For this analysis, we excluded 20 individuals for whom no serum sample was available. MEMO was approved by the ethics committee of the University of Münster, Germany; all individuals provided written informed consent.

### Definition of exposure

To measure the IgG-antibody titers against pre-defined pathogens responsible for chronic infections, we used the “*Helicobacter pylori* IgG ELISA” (IBL International, (REF) RE56381) (cut-off index for a positive result of > 1.2), the “*Toxoplasma gondii* IgG ELISA” (IBL International, (REF) RE58371) (cut-off index for a positive result of > 1.2), and the “*Borrelia burgdorferi s.l.* Europe plus TpN17 LINE IgG Line Immunoblot” (Virotech diagnostics, (REF) WE225G32). We also measured CMV IgG-antibody titers (cut-off for positive > = 6 AU/ml) with Architect-CMV-IgG (Abbott-Diagnostics, (REF) 6C1530) and EBV IgG-antibody titers (signal to cut-off ratio for positive > = 1) with Architect-EBV-VCA-IgG (Abbott-Diagnostics, (REF) 3P6525), HHV-6 IgG-antibody titers (signal to cut-off ratio for positive > 1.1 and for seroborderline > = 0.9 to < = 1.1) with Anti-HHV-6-IgG ELISA-VIDITEST (VIDIA, (REF) ODZ-235), and HSV-1/2 IgG-antibody titers (cut-off for positive > 1.1) with LIASION-HSV-1/2 IgG (DiaSorin S.p.A., (REF) 310,800), according to the manufacturer’s instructions. For the primary analysis, we dichotomized all antibody titers into seropositive or seronegative, according to the manufacturers’ manual. However, *B. burgdorferi s.l.* and HHV-6 were, according to the manual, trichotomized into seropositive, seronegative, and seroborderline (when the measurement was between two threshold values). We considered individuals seroborderline for *B. burgdorferi s.l.* or HHV-6 as seronegative in the primary analysis. Based on the dichotomized serostatus, we calculated an infection sum score to examine the cumulative effect of seropositivity for each individual. This score consisted of values from two to seven; no individual was seropositive for zero or only one infectious agent. We performed sensitivity analyses in which we considered the individuals seroborderline for *B. burgdorferi s.l.* and HHV-6 as seropositive. Moreover, we constructed separate cumulative scores for viruses and bacteria/protozoa using the same approach. In an additional sensitivity analysis, we divided CMV, *H. pylori*-, and *B. burgdorferi s.l*. antibody titers into quartiles for a better comparison with the previous literature.

### Definition of outcome

We defined all-cause mortality as derived by official cause of death certificates from local health departments as the outcome of interest. Four mortality follow-ups were performed since the initiation of MEMO (latest December 2015). Individuals, who were not reported to be dead, were censored at the time of the last mortality assessment, if they were not lost to follow up.

### Definition of covariables

Using the disjunctive cause criterion (VanderWeele and Shpitser 2011) for confounder identification, we included age (in years), sex, socioeconomic status (SES) (Donnelly et al. 2018), history of stroke (Chamorro et al. 2007), any kind of cancer (Rolston 2017), myocardial infarction (Truffa et al. 2012), diabetes mellitus type 1 or 2 (Casqueiro et al. 2012), smoking status (Bagaitkar et al. 2008), BMI (kg/m^2^; with BMI > 30 defined as obese according to WHO definition (Dobner and Kaser 2018; WHO 2019)), and hypertension as potential confounders (Gu et al. 2017). All potential confounders were assessed as self-reported variables at baseline only, using a standardized interview. Due to the limited information available in MEMO, we used the number of education years (including apprenticeships) as a surrogate for SES. We considered individuals who had not reported a comorbidity as not having the respective comorbidity in the primary analysis. Given the limited sample size of the study, we adapted the approach of Diederichs et al. to the field of time to event analyses to calculate a weighted comorbidity score (based on cox proportional hazard regression) in order to keep the model parsimonious (Diederichs et al. 2012).

### Statistical methods

We calculated the accelerated failure time (AFT) and its 95% confidence interval (CI) in a parametric survival model based on a Weibull distribution assumption to describe the effect of serostatus on all-cause mortality. We assessed the constant hazard assumption graphically. We used parametric survival models because constant hazards of death could be assumed, whereas the proportional hazards assumption crucial for semi-parametric survival models was not fulfilled in many of the models to be performed. We first calculated AFT in univariable analyses including serostatus only, as well as in multivariable analyses adjusted for age (continuous), sex (binary), number of education years (continuous), and the comorbidity score (continuous, ranging from 0 to 2.5). In a second step, we used the cumulative infection score (both continuously and categorically, to assess the functional form of a possible effect) instead of single infections in the univariable and the adjusted multivariable analysis. In the sensitivity analysis based on separate scores for viruses and bacteria/protozoa as well as in the sensitivity analyses based on quartiles of the CMV-, *H. pylori*-, and *B. burgdorferi s.l*. IgG antibody titer distribution, we used the same analysis approach as for the cumulative infection score. AFTs (where the null hypothesis of no effect is represented by 0, and effects are represented as a relative decrease or increase in survival time in percent) were also transformed into hazard ratios (HR, where no effects corresponds to a HR of 1) for better comparison with the literature. We conducted all analyses using the package “survival” (Therneau 2015) in R version 3.6.1.

## Results

**Fig. 1:**
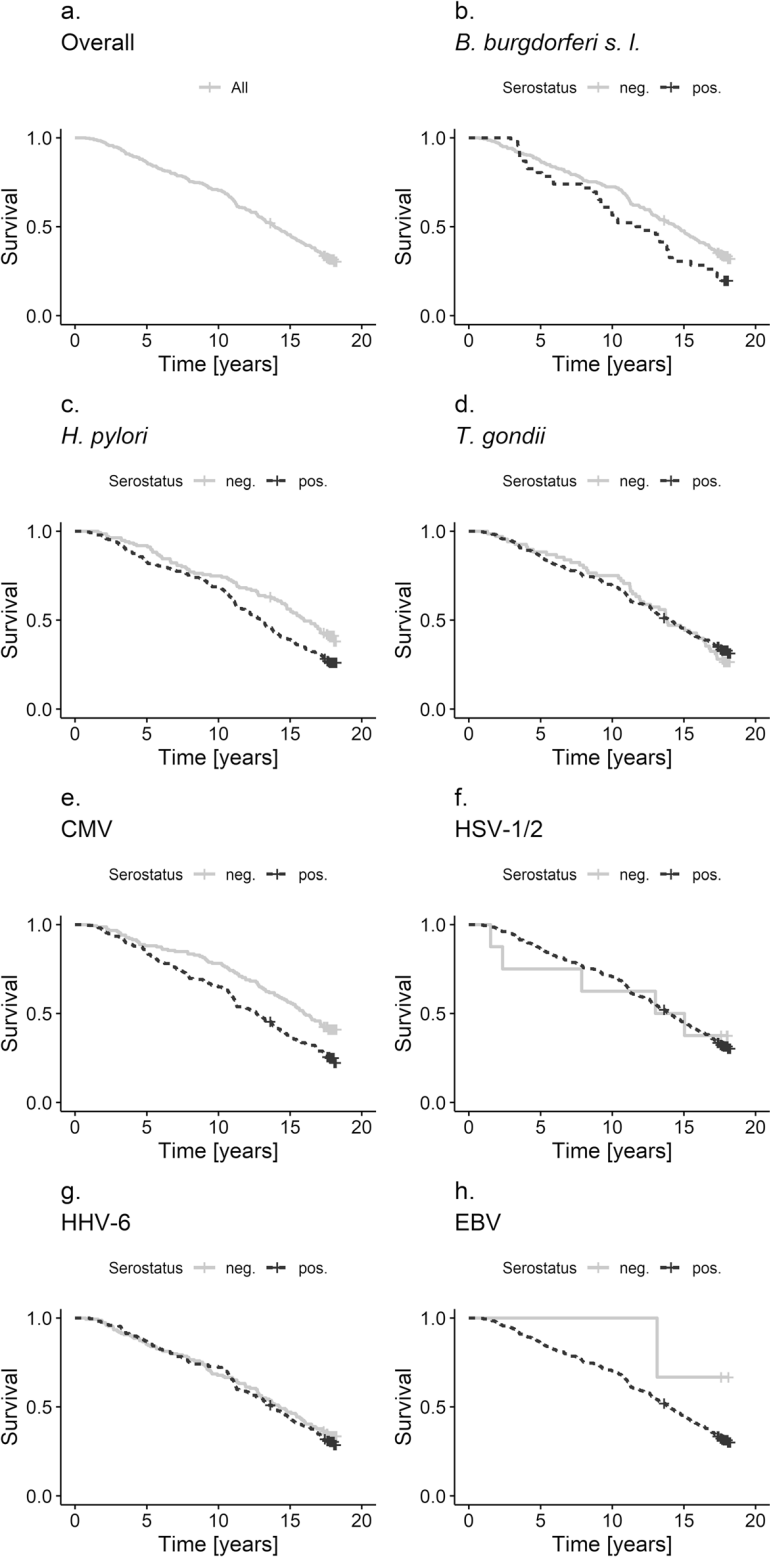
Kaplan Meier plots showing overall survival **(a)** and the observed effect of seropositivity of seven chronic infections (**b**–**h**) on all-cause mortality in MEMO (*n* = 365)

**Fig. 2:**
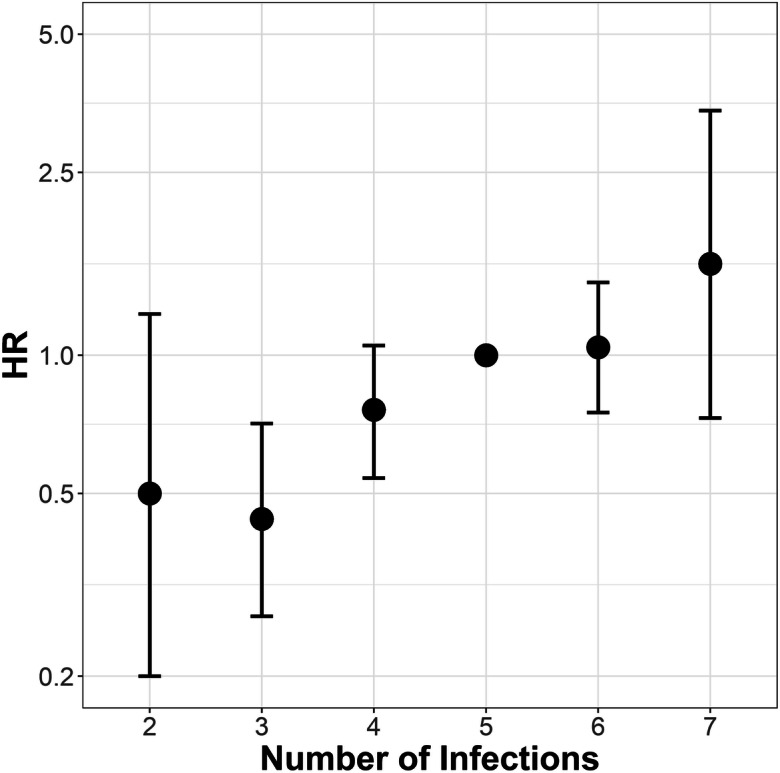
Visualization of the association between all-cause mortality and the cumulative number of infections an individual was seropositive for (*n* = 365; five as reference). Displayed are effect estimates for hazard ratios with 95% Confidence intervals

**Fig. 3:**
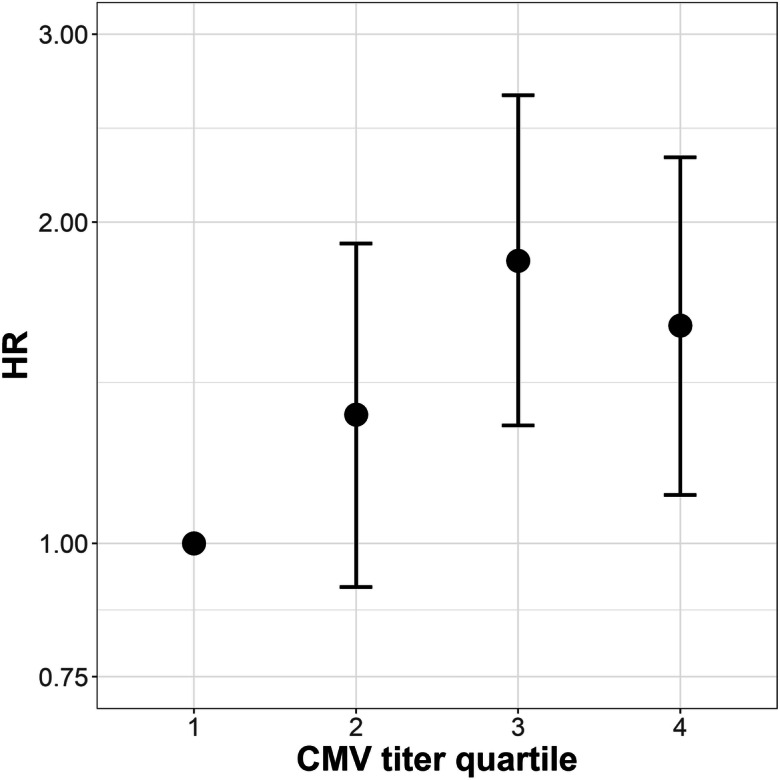
Visualization of the association between all-cause mortality and CMV IgG antibody titer quartiles (*n* = 365; first as reference). Displayed are effect estimates for hazard ratios with 95% confidence intervals

The 365 study participants had a median age of 73 years (interquartile range 69–76) at baseline; 46% were women (Table 1). Of all participants 250 died during follow-up (68%); median survival time until the last vital status follow-up was 14 years. Seropositivity in this study varied between 13% for *B. burgdorferi s.l.* and 99% for EBV. Females were more likely to be seropositive for CMV and HHV-6, but less likely to be seropositive for *H. pylori* and *B. burgdorferi s.l.* (Supplementary Table 4)*.* Moreover, being seropositive for CMV and HHV-6 was associated with a body mass index of 30 or above (Supplementary Table 4). Among the seven chronic infections investigated, reduced survival time was observed for those seropositive for *H. pylori* (AFT − 15.92, 95% CI − 29.96; − 1.88), CMV (AFT − 22.81, 95% CI − 36. 42; −9.22), and *B. burgdorferi s.l.* (AFT − 25.25, 95% CI − 43.40; − 7.10) after adjusting for potential confounders (Table 2). We did not find evidence for an effect of seropositivity for EBV, HSV-1/2, *T. gondii*, or HHV-6 on overall survival (Fig. 1). However, confidence intervals were wide for EBV and HSV-1/2 given the low number of seronegative individuals. The cumulative infection score showed a clear association with reduced survival in the multivariable analysis (AFT − 12.42, 95% CI − 18.55; − 6.30, per additional score point; Fig. 2 and Table 3). This was consistent across all age groups (Supplementary Table 9). Results for separate scores for viruses and bacteria/protozoa were in line with the main analyses. When we considered seroborderline results as seropositive, seropositivity for *B. burgdorferi s.l.* was no longer as strongly associated with reduced survival time as in the primary analysis (AFT − 9.66, 95% CI − 25.21; 5.88) (Supplementary Table 1). Increasing CMV antibody titers (when divided in quartiles) were associated with reduced survival in the multivariable analysis (AFT − 8.53, 95% CI − 14.14; − 2.93, per quartile rising, Fig. 3 and Table 4), while there was no difference between the upper three antibody titer quartiles for *B. burgdorferi s.l*. and *H. pylori.* All additional sensitivity analyses led to qualitatively unchanged results when compared to the primary analysis (Supplementary Table 1–9).

**Table 1:** Baseline characteristics of study participants (*n* = 365)

Demographic characteristics	Study population
Female, *n* (%)	168 (46%)
Age, median, min to max	73, 65–83
Number of education years, median, min to max	10, 8–17
Follow-up years, median, 1st and 3rd quartile	14, 8–18
Comorbidities and risk factors	Present
Body mass index > 30, *n* (%)	88 (24%)
History of stroke, *n* (%)	24 (7%)
History of any cancer, *n* (%)	13 (4%)
History of myocardial infarction, *n* (%)	33 (9%)
Diabetes mellitus, *n* (%)	39 (11%)
Smoking, *n* (%)	186 (51%)
Hypertension, *n* (%)	173 (47%)
Serostatus for selected pathogens	Seropositive
*B. burgdorferi s.l.*, *n* (%) Seroborderline for *B. burgdorferi s.l.*, *n* (%)	46 (13%) 30 (8%)
CMV, *n* (%)	214 (59%)
HHV-6, *n* (%) Seroborderline for HHV-6, *n* (%)	216 (59%) 23 (6%)
*H. pylori*, *n* (%)	230 (63%)
*T. gondii*, *n* (%)	297 (81%)
HSV-1/2, *n* (%)	357 (98%)
EBV, *n* (%)	362 (99%)
Cumulative infection score, median, min to max	5, 2–7

**Table 2:** Association between seropositivity for selected chronic infections and all-cause mortality (*n* = 365)

			Univariable analysis		Multivariable analysis*	
Infectious agent	Deaths	Follow-up years	AFT in % (95%-CI)	HR (95%-CI)	AFT in % (95%-CI)	HR (95%-CI)
*H. pylori*						
Seropositive (*n* = 230)	170	2777	− 22.32 (− 37.45; − 7.19)	1.48 (1.14; 1.94)	− 15.92 (− 29.96; − 1.88)	1.36 (1.04; 1.79)
Seronegative (*n* = 135)	80	1830	0	1	0	1
*T. gondii*						
Seropositive (*n* = 297)	200	3730	3.44 (− 14.24; 21.12)	0.94 (0.69; 1.28)	− 1.34 (− 17.44; 14.76)	1.03 (0.75; 1.40)
Seronegative (*n* = 68)	50	877	0	1	0	1
*B. burgdorferi s.l.* **						
Seropositive (*n* = 46)	37	522	− 21.52 (− 41.41; − 1.63)	1.46 (1.03; 2.07)	− 25.25 (− 43.4; − 7.10)	1.64 (1.15; 2.35)
Seronegative (*n* = 319)	213	4085	0	1	0	1
CMV						
Seropositive (*n* = 214)	161	2531	− 26.37 (− 41.11; − 11.63)	1.60 (1.23; 2.07)	− 22.81 (− 36.41; − 9.22)	1.56 (1.20; 2.03)
Seronegative (*n* = 151)	89	2076	0	1	0	1
HHV-6 **						
Seropositive (*n* = 216)	151	2714	− 4.25 (− 18.71; 10.21)	1.08 (0.84; 1.39)	− 5.22 (− 18.38; 7.94)	1.11 (0.86; 1.43)
Seronegative (*n* = 149)	99	1893	0	1	0	1
HSV-1/2						
Seropositive (*n* = 357)	245	4514	− 1.79 (− 52.32; 48.74)	1.03 (0.43; 2.50)	− 28.93 (− 75.02; 17.16)	1.75 (0.72; 4.29)
Seronegative (*n* = 8)	5	93	0	1	0	1
EBV						
Seropositive (*n* = 362)	249	4558	− 61.02 (− 173.12; 51.09)	2.92 (0.41; 20.78)	− 34.96 (− 137.38; 67.45)	1.97 (0.27; 14.30)
Seronegative (*n* = 3)	1	49	0	1	0	1

*adjusted for age, sex, education years and a comorbidity index

**seroborderline as seronegative

**Table 3:** Association between cumulative infection score and all-cause mortality (*n* = 365)

			Univariable analysis		Multivariable analysis*	
Infectious agents	Deaths	Follow-up years	AFT in % (95%-CI)	HR (95%-CI)	AFT in % (95%-CI)	HR (95%-CI)
Cumulative infection score (linear effect)	250	4607	− 13.72 (− 20.64; − 6.80)	1.28 (1.13; 1.44)	− 12.42 (− 18.55; − 6.30)	1.28 (1.13; 1.44)
Cumulative number of infections a person was seropositive for						
*2 Infections (n = 9)*	5	117	22.08 (− 28.29; 72.45)	0.67 (0.28; 1.65)	34.75 (− 10.63; 80.13)	0.5 (0.20; 1.23)
*3 Infections (n = 38)*	20	575	33.88 (6.89; 60.87)	0.55 (0.34; 0.88)	41.57 (17.06; 66.07)	0.44 (0.27; 0.71)
*4 Infections (n = 91)*	54	1207	17.91 (− 55.32; 36.37)	0.73 (0.52; 1.01)	14.15 (− 2.46; 30.76)	0.76 (0.54; 1.05)
5 Infections** (n = 145)	106	1782	0	1	0	1
*6 Infections (n = 73)*	58	831	− 12.71 (− 30.68; 5.26)	1.25 (0.91; 1.73)	− 2.14 (− 18.50; 14.23)	1.04 (0.75; 1.44)
*7 Infections (n = 9)*	7	94	− 18.0 (− 60.94; 24.94)	1.38 (0.64; 2.96)	− 23.03 (− 61.89; 15.84)	1.58 (0.73; 3.41)

*adjusted for age, sex, education years and a comorbidity index;

**five infections as reference category

**Table 4:** Association between CMV IgG antibody titer quartiles and all-cause mortality (*n* = 365)

			Univariable analysis		Multivariable analysis*	
CMV quartile	Deaths	Follow-up years	AFT in % (95%-CI)	HR (95%-CI)	AFT in % (95%-CI)	HR (95%-CI)
CMV quartiles (linear effect)	250	4607	− 9.11 (− 15.28; − 2.94)	1.17 (1.05; 1.31)	− 8.53 (− 14.14; − 2.93)	1.18 (1.06; 1.32)
Quartiles						
First (0.0–0.6 AU/ml)**	56	1335	0	1	0	1
*Second (0.7–116.7 AU/ml)*	58	1145	− 11.34 (− 32.08; 9.40)	1.22 (0.84; 1.77)	− 14.19 (− 33.06; 4.69)	1.32 (0.91; 1.91)
*Third (116.8–210 AU/ml)*	70	1037	− 32.07 (− 52.03; − 12.10)	1.77 (1.24; 2.51)	− 31.09 (− 49.36; −12.83)	1.84 (1.29; 2.63)
*Fourth (210.1–250 AU/ml)*	66	1090	− 24.29 (− 44.46; − 4.12)	1.54 (1.08; 2.2)	− 23.90 (− 42.32; − 5.49)	1.60 (1.11; 2.23)

*adjusted for age, sex, education years and a comorbidity index;

**first quartile as reference (0–0.6 AU/ml)

## Discussion

Our results indicate that community dwelling individuals older than 65 years seropositive for *H. pylori*, *B. burgdorferi s.l.*, or CMV have a reduced overall survival time compared with seronegative individuals after adjustment for potential confounders.

Within our study, CMV seropositivity had the largest individual effect on all-cause mortality (HR: 1.56, 95% CI 1.20; 2.03). This confirms results of three previous cohort studies, which all reported decreased survival time in individuals seropositive for CMV. A study from the UK focused as well on elderly individuals (mean age 74), and reported an HR of 1.35 (95% CI 1.04; 1.75) (Savva et al. 2013) which is in line with the effect estimate in our study. An analysis based on NHANES III (National Health and Nutrition Examination Survey III 1988–1994) data from the USA reported a smaller effect size of CMV seropositivity on all-cause mortality than our study with an HR of 1.19 (95% CI 1.01; 1.41). The mean age in NHANES was, however, considerably lower than in our study with 51 years for seropositive and 41 years for seronegative individuals at baseline (Simanek et al. 2011). Assuming that most individuals were infected approximately at the same age in the NHANES III and in our study, this may imply that a longer exposure time to the chronic infectious agent results in a cumulative adverse effects. Alternatively, the difference in effect size between our population and the one in the younger NHANES III cohort may imply that adverse CMV effects are more pronounced in older people. CMV may prompt a stronger inflammatory response, which, superposed with the natural age-related increase in inflammation in the organism, may increase an individual’s vulnerability for NCDs and its risk of dying earlier (De Martinis et al. 2005). This is supported by our recent experimental study in an animal model of CMV infection that has shown that latent CMV infection of fat tissue induces inflammatory responses in aged mice (Contreras et al. 2019). We found in our study indeed an association between CMV seropositivity and obesity but unlike a recent meta-analysis (Wang et al. 2017) no increased risk for cardiovascular diseases. Future studies will need to explore the interaction of CMV infection and inflammation in elderly populations in more detail. A study based on the Women’s Health and Aging Studies I and II (Wang et al. 2010) in the USA, which focused on female individuals with a mean age of 74 years, used IgG antibody titer quintiles, and found clear evidence for a reduced survival in the highest (HR 2.79 95% CI 1.22; 6.40) compared to the first quintile. The effect estimate is considerably higher than the one we obtained for the highest quartile in our study (HR 1.60 95% CI 1.11; 2.23); however, confidence intervals of both studies overlap (Supplementary Figure 1).

We also found a reduced overall survival for individuals seropositive for *H. pylori* with an adjusted HR of 1.36 (95% CI 1.04; 1.79). Contrary to our findings, a study from 2013 based on NHANES III data with 9895 participants did not find an association between *H. pylori* seropositivity and all-cause mortality (HR 1.00, 95% CI 0.84; 1.18) (Chen et al. 2013). Analogous to CMV, the longer exposure time to the infectious agent in the elderly might lead to an accelerated immunosenescence and greater inflammation (De Martinis et al. 2005). Unlike for CMV, our data did not suggest an effect of *H. pylori* IgG antibody titer on mortality.

Individuals seropositive for *B. burgdorferi s.l.* had also a reduced survival time in the multivariable analysis (HR 1.64, 95% CI 1.15; 2.35) of our study. This effect, however, was reduced considerably when we classified seroborderline individuals as seropositive (HR 1.21, 95% CI 0.89; 1.63). To our knowledge, there is no other study, which assessed an association between *B. burgdorferi s.l.* serostatus and all-cause mortality; thus, our study adds new evidence to the literature, which needs to be validated in other population-based cohort studies with long-term follow-up. Unlike for CMV, our data did not suggest an effect of *B. burgdorferi s.l.* IgG antibody titer on mortality.

When we combined the investigated antibody titers into a cumulative infection score, we found evidence for a dose-response effect (HR 1.28, 95% CI 1.13; 1.44; per additional score point). Simanek and colleagues built a categorical infection score based on four antibody titers (CMV, HSV-1/2, and *H. pylori*), and found much stronger effect sizes in univariable models than our study did, reaching from an HR of 2.5 when being seropositive for one infection up to an HR of 7.9 when being seropositive for four infections (Simanek et al. 2015). In their adjusted multivariable model, this effect size was more similar to our results, but did no longer show a linear increase with increasing numbers of infections, while our study still does. The linear effect in our study can be explained in the way that every single infection induces a perpetual stimulation of the immune system which then leads to an additive effect (O'Connor et al. 2006). This might correspond to a higher rate of necroptosis, which has been reported in the elderly (Royce et al. 2019), and to an increased release of damage-associated molecule patters (DAMPs), which induce inflammation and might lead to a feedback loop. A second potential explanation for the overall increased mortality of those seropositive for several infections is that a high cumulative infection score is a proxy for a failing immune system as present in symptomatic or subclinical immunodeficiency. Immunodeficiencies can be divided into primary immunodeficiencies, the intrinsic malfunctioning of the immune system (Snydman et al. 2014), and secondary immunodeficiencies, which are caused by extrinsic factors like malnutrition or cancer (Chinen and Shearer 2010). Since the defective immune system cannot fulfill its role as a defender, affected individuals are especially susceptible to infectious diseases (Rezaei et al. 2011), so that infections are a major cause for mortality in individuals with an immunodeficiency (Stein et al. 1992). Contrary to the explanation based on the immunosenescence hypothesis, seropositivity for chronic infections would in this case not fulfill the definition of a cause of all-cause mortality, but would rather be a mediator on the causal pathway between immunodeficiency and all-cause mortality.

Seropositivity varied considerably between the different pathogens in our study. While this had an impact on the interpretability of results for highly prevalent pathogens, seropositivity values were generally in line with previous literature (Franck et al. 2017; Korr et al. 2017; Lachmann et al. 2018; Pleyer et al. 2019).

## Limitations

Our study was based on serum IgG-antibody titers only, but did not have information on the clinical manifestation of the infection, the presence of symptoms or the timing of the exposure to the infectious agent. Therefore, the observed effect is difficult to interpret from a public health perspective. Moreover, our study was not large enough to provide sufficient power to assess the effect of highly prevalent infections like EBV and HSV-1/2; therefore, the results for these viruses are not conclusive. The handling of seroborderline results in the primary analysis might have biased the results. While most analyses were not affected in our sensitivity analysis, the 95% CI for the HR included 1 when counting seroborderline individuals as seropositive in the case of *B. burgdorferi s.l.* We used education years as a proxy for socioeconomic status although they cover only part of what socioeconomic status means. However, no additional information was available in the underlying cohort study.

## Conclusion

Our study showed a reduced survival time for individuals seropositive for CMV, *H. pylori*, and *B. burgdorferi s.l*. Moreover, we found a linear effect of the number of infections an individual was seropositive for on all-cause mortality. From a public health perspective, vaccination against the causes of chronic infections might extend the overall survival time for the elderly, if seropositivity for chronic infections is indeed causally linked to all-cause mortality. Vaccines against CMV and *B. burgdorferi s.l.* are already in development (Comstedt et al. 2017; La Rosa et al. 2017), whereas a vaccine against *H. pylori* seems less likely to be available in the nearer future (Sutton and Boag 2018). Another preventive strategy could be screening and antibiotic treatment for *H. pylori* colonization (Kim et al. 2015*)*, irrespective of the clinical manifestation. Experimental approaches unspecific to individual pathogens, like mTOR inhibitors, have been shown to prevent senescence in model organisms (Nacarelli et al. 2018) and in first human trials (Chung et al. 2019) and might become a preventive option for the future.

## Electronic supplementary material

ESM 1 (DOCX 44 kb)

Fig. 4 Kaplan Meier survival curve by levels of the cumulative infection score, based on the sum of seropositive results (among CMV, HSV-1/2, HHV-6, EBV*, H. pylori*, *T. gondii*, and *B. burgdorferi s. l.*) (PNG 49.7 kb)

High resolution image (TIF 134 kb)
