# Supplementary material for: Seropositivity for pathogens associated with chronic infections is a risk factor for all-cause mortality in the elderly: findings from the Memory and Morbidity in Augsburg Elderly (MEMO) Study
Source: GeroScience. 2020 Jul 9;42(5):1365–76. doi: 10.1007/s11357-020-00216-x (PMC7525922; doi:10.1007/s11357-020-00216-x)
Supplement: Supplementary file 1 — (DOCX 44 kb) [file 11357_2020_216_MOESM1_ESM.docx]

## Supplementary Analysis

**Supplementary table 1** Sensitivity analysis of the association between seroborderline infections and all-cause mortality when counting seroborderline as seropositive (n=365)

|  |  |  | **Univariable analysis** | | **Multivariable analysis*** | |
| --- | --- | --- | --- | --- | --- | --- |
| **Infectious agent** | **Events** | **Follow-up years** | **AFT in % (95%-CI)** | **HR (95%-CI)** | **AFT in % (95%-CI)** | **HR (95%-CI)** |
| HHV-6 |  |  |  |  |  |  |
| Seropositive (n=239) | 166 | 3029 | -1.90 (-16.87; 13.07) | 1.03 (0.8; 1.34) | -4.89 (-18.50; 8.72) | 1.1 (0.84; 1.43) |
| Seronegative (n=126) | 84 | 1578 | 0 | 1 | 0 | 1 |
| *B. burgdorferi s.l.* |  |  |  |  |  |  |
| Seropositive (n=76) | 55 | 924 | -8.30 (-25.37; 8.77) | 1.16 (0.86; 1.56) | -9.66 (-25.21; 5.88) | 1.21 (0.89; 1.63) |
| Seronegative (n=289) | 195 | 3683 | 0 | 1 | 0 | 1 |

*adjusted for age, sex, education years and a comorbidity index

**Supplementary Table 2** Association between seropositivity for selected chronic infections (with all infectious in one model) and all-cause mortality (n=365)

|  | **Multivariable analysis (all infections in one model)** | | **Multivariable analysis (all infections in one model adjusted for potential confounders*)** | |
| --- | --- | --- | --- | --- |
| **Infectious agent** | **AFT in % (95%-CI)** | **HR (95%-CI)** | **AFT in % (95%-CI)** | **HR (95%-CI)** |
| *H. pylori* | -19.81 (-35.2; -4.42) | 1.43 (1.08; 1.88) | -11.63 (-25.81; 2.55) | 1.26 (0.95; 1.67) |
| *T. gondii* | 3.89 (-13.51; 21.29) | 0.93 (0.68; 1.27) | 1.5 (-14.31; 17.3) | 0.97 (0.71; 1.33) |
| *B. burgdorferi s.l.* | -19.67 (-39.27; 0.07) | 1.42 (1; 2.02) | -23.11 (-41.04; -5.17) | 1.59 (1.11; 2.27) |
| CMV | -23.34 (-38.14; -8.54) | 1.52 (1.17; 1.98) | -22.52 (-36.23; -8.82) | 1.57 (1.19; 2.06) |
| HHV-6 | -5.21 (-19.46; 9.04) | 1.1 (0.85; 1.42) | -3.18 (-16.15; 9.77) | 1.07 (0.82; 1.38) |
| HSV-1/2 | 11.57 (-38.53; 62.67) | 0.81 (0.33; 2) | -21.84 (-67.07; 23.4) | 1.55 (0.63; 3.82) |
| EBV | -56.29 (-166.29; 53.71) | 2.75 (0.38; 19.7) | -33.29 (133.12; 66.54) | 1.94 (0.26; 14.28) |

*adjusted for age, sex, education years and a comorbidity index

**Supplementary Table 3** Frequency of individual combinations of positive serostatuses in study participants excluding EBV and HSV-1/2 (n=365)

| **Combination of positive serostatuses** | **Number** |
| --- | --- |
| *H. pylori*, *T. gondii*, *B. burgdorferi s.l.*, CMV, HHV-6 | 9 |
| *H. pylori*, *T. gondii*, *B. burgdorferi s.l.*, CMV | 5 |
| *H. pylori*, *T. gondii*, *B. burgdorferi s.l.*, HHV-6 | 3 |
| *H. pylori*, *T. gondii*, CMV, HHV-6 | 57 |
| *H. pylori*, *B. burgdorferi s.l.*, CMV, HHV-6 | 2 |
| *T. gondii*, *B. burgdorferi s.l.*, CMV, HHV-6 | 3 |
| *H. pylori*, *T. gondii*, *B. burgdorferi s.l.*, | 6 |
| *H. pylori*, *T. gondii*, CMV | 31 |
| *H. pylori*, *T. gondii*, HHV-6 | 29 |
| *H. pylori*, *B. burgdorferi s.l.*, CMV | 1 |
| *H. pylori*, *B. burgdorferi s.l.*, HHV-6 | 3 |
| *H. pylori*, CMV, HHV-6 | 12 |
| *T. gondii*, *B. burgdorferi s.l.*, CMV | 0 |
| *T. gondii*, *B. burgdorferi s.l.*, HHV-6 | 6 |
| *T. gondii*, CMV, HHV-6 | 35 |
| *B. burgdorferi s.l.*, CMV, HHV-6 | 0 |
| *H. pylori*, *T. gondii*, | 20 |
| *H. pylori*, *B. burgdorferi s.l.* | 1 |
| *H. pylori*, CMV | 7 |
| *H. pylori*, HHV-6 | 5 |
| *T. gondii*, *B. burgdorferi s.l.*, | 1 |
| *T. gondii*, CMV | 12 |
| *T. gondii*, HHV-6 | 24 |
| *B. burgdorferi s.l.*, CMV | 1 |
| *B. burgdorferi s.l.*, HHV-6 | 0 |
| CMV, HHV-6 | 7 |

**Supplementary Table 4** Frequency of demographic factors, comorbidities and risk factors, stratified by serostatus for any of the pathogens studies (n=365)

| ***B. burgdorferi s.l.*** | **Seropositive (n=46)** | **Seronegative (n=319)** |
| --- | --- | --- |
| Female, n (%) | 16 (34.78 %) | 152 (47.65 %) |
| Age, median, min to max | 74, 65-83 | 73, 65-83 |
| Number of education years, median, min to max | 10, 8-17 | 10, 8-17 |
| Body mass index > 30, n (%) | 8 (17.39 %) | 80 (25.09 %) |
| History of stroke, n (%) | 3 (6.52 %) | 21 (6.58 %) |
| History of any cancer, n (%) | 2 (4.35 %) | 11 (3.45 %) |
| History of myocardial infarction, n (%) | 7 (15.22 %) | 26 (8.15 %) |
| Diabetes mellitus, n (%) | 6 (13.04 %) | 33 (10.34 %) |
| Smoking, n (%) | 21 (45.65 %) | 165 (51.72 %) |
| Hypertension, n (%) | 16 (34.78 %) | 157 (49.22 %) |
|  |  |  |
| **CMV** | **Seropositive (n=214)** | **Seronegative (n=151)** |
| Female, n (%) | 106 (49.53 %) | 62 (41.06 %) |
| Age, median, min to max | 73, 65-83 | 72, 65-83 |
| Number of education years, median, min to max | 10, 8-17 | 10, 8-17 |
| Body mass index > 30, n (%) | 64 (29.91 %) | 24 (15.89 %) |
| History of stroke, n (%) | 15 (7 %) | 9 (5.96 %) |
| History of any cancer, n (%) | 7 (3.27 %) | 6 (3.97 %) |
| History of myocardial infarction, n (%) | 21 (9.81 %) | 12 (7.95 %) |
| Diabetes mellitus, n (%) | 29 (13.55 %) | 10 (6.62 %) |
| Smoking, n (%) | 114 (53.27 %) | 72 (47.68 %) |
| Hypertension, n (%) | 101 (47.20 %) | 72 (47.68 %) |
|  |  |  |
| **HHV-6** | **Seropositive (n=216)** | **Seronegative (n=149)** |
| Female, n (%) | 111 (51.39 %) | 57 (38.26 %) |
| Age, median, min to max | 73, 65-83 | 73, 65-82 |
| Number of education years, median, min to max | 10, 8-17 | 10, 8-17 |
| Body mass index > 30, n (%) | 61 (28.24 %) | 27 (18.12 %) |
| History of stroke, n (%) | 13 (6.02 %) | 11 (7.38 %) |
| History of any cancer, n (%) | 7 (3.24 %) | 6 (4.02 %) |
| History of myocardial infarction, n (%) | 15 (6.94 %) | 18 (12.08 %) |
| Diabetes mellitus, n (%) | 23 (10.65 %) | 16 (10.73 %) |
| Smoking, n (%) | 108 (50 %) | 78 (52.35 %) |
| Hypertension, n (%) | 101 (46.76 %) | 72 (48.32 %) |
|  |  |  |
| ***H. pylori*** | **Seropositive (n=230)** | **Seronegative (n=135)** |
| Female, n (%) | 90 (39.12 %) | 78 (57.78 %) |
| Age, median, min to max | 73, 65-83 | 73, 65-83 |
| Number of education years, median, min to max | 10, 8-17 | 10, 8-17 |
| Body mass index > 30, n (%) | 61 (26.52 %) | 27 (20 %) |
| History of stroke, n (%) | 13 (5.65 %) | 11 (8.15 %) |
| History of any cancer, n (%) | 9 (3.91 %) | 4 (2.96 %) |
| History of myocardial infarction, n (%) | 23 (10 %) | 10 (7.41 %) |
| Diabetes mellitus, n (%) | 28 (12.17 %) | 11 (8.15 %) |
| Smoking, n (%) | 125 (54.35 %) | 61 (45.19 %) |
| Hypertension, n (%) | 104 (45.22 %) | 69 (51.11 %) |
|  |  |  |
| ***T. gondii*** | **Seropositive (n=297)** | **Seronegative (n=68)** |
| Female, n (%) | 143 (48.15 %) | 25 (36.76 %) |
| Age, median, min to max | 73, 65-83 | 73, 65-81 |
| Number of education years, median, min to max | 10, 8-17 | 10, 8-17 |
| Body mass index > 30, n (%) | 76 (25.59 %) | 12 (17.65 %) |
| History of stroke, n (%) | 19 (6.4 %) | 5 (7.35 %) |
| History of any cancer, n (%) | 11 (3.7 %) | 2 (2.94 %) |
| History of myocardial infarction, n (%) | 25 (8.41 %) | 8 (11.76 %) |
| Diabetes mellitus, n (%) | 30 (10.1 %) | 9 (13.24 %) |
| Smoking, n (%) | 154 (51.85 %) | 32 (47.06 %) |
| Hypertension, n (%) | 133 (44.78 %) | 40 (58.82 %) |
|  |  |  |
| **HSV-1/2** | **Seropositive (n=357)** | **Seronegative (n=8)** |
| Female, n (%) | 165 (46.22 %) | 3 (37.5 %) |
| Age, median, min to max | 73, 65-83 | 77, 66-82 |
| Number of education years, median, min to max | 10, 8-17 | 12, 10-17 |
| Body mass index > 30, n (%) | 86 (24.09 %) | 2 (25 %) |
| History of stroke, n (%) | 24 (6.72 %) | 0 (0 %) |
| History of any cancer, n (%) | 12 (3.36 %) | 1 (12.5 %) |
| History of myocardial infarction, n (%) | 31 (8.68 %) | 2 (25 %) |
| Diabetes mellitus, n (%) | 39 (10.92 %) | 0 (0%) |
| Smoking, n (%) | 181 (50.7 %) | 5 (62.5 %) |
| Hypertension, n (%) | 167 (46.78 %) | 6 (75 %) |
|  |  |  |
| **EBV** | **Seropositive (n=362)** | **Seronegative (n=3)** |
| Female, n (%) | 165 (45.58 %) | 3 (100 %) |
| Age, median, min to max | 73, 65-83 | 73, 72-74 |
| Number of education years, median, min to max | 10, 8-17 | 10, 10-17 |
| Body mass index > 30, n (%) | 88 (24.31 %) | 0 (0 %) |
| History of stroke, n (%) | 23 (6.35 %) | 1 (33.33 %) |
| History of any cancer, n (%) | 13 (3.59 %) | 0 (0 %) |
| History of myocardial infarction, n (%) | 33 (9.12 %) | 0 (0 %) |
| Diabetes mellitus, n (%) | 39 (10.77 %) | 0 (0 %) |
| Smoking, n (%) | 186 (51.38 %) | 0 (0 %) |
| Hypertension, n (%) | 173 (47.79 %) | 0 (0 %) |

**Supplementary Table 5** Association between cumulative virus score and all-cause mortality (n=365)

|  |  |  | **Univariable analysis** | | **Multivariable analysis*** | |
| --- | --- | --- | --- | --- | --- | --- |
| **Infectious agents** | **Deaths** | **Follow-up years** | **AFT in % (95%-CI)** | **HR (95%-CI)** | **AFT in % (95%-CI)** | **HR (95%-CI)** |
| Cumulative virus score  (linear effect) | 250 | 4607 | -14.4 (-24.12; -4.68) | 1.29 (1.09; 1.53) | -13.7 (-22.32; -5.09) | 1.31 (1.11; 1.55) |
| Cumulative number of virus infections a person was seropositive for |  |  |  |  |  |  |
| *1 Infections (n=2)* | 1 | 31 | 14.94 (-97.33; 127.21) | 0.77 (0.11; 5.59) | 10.73 (-91.01; 112.46) | 0.81 (0.11; 5.96) |
| *2 Infections** (n=70)* | 40 | 978 | 0 | 1 | 0 | 1 |
| *3 Infections (n=165)* | 111 | 2063 | -16.99 (-37.51; 3.53) | 1.35 (0.94; 1.94) | -28.04 (-46.89; -9.19) | 1.73 (1.2; 2.5) |
| *4 Infections (n=128)* | 98 | 1535 | -56.96 (-77.89; -36.03) | 1.69 (1.17; 2.44) | -31.75 (-51.02; -12.49) | 1.86 (1.28; 2.72) |

*adjusted for age, sex, education years and a comorbidity index; **two infections as reference category

**Supplementary Table 6** Association between cumulative bacteria and protozoa score and all-cause mortality (n=365)

|  |  |  | **Univariable analysis** | | **Multivariable analysis*** | |
| --- | --- | --- | --- | --- | --- | --- |
| **Infectious agents** | **Deaths** | **Follow-up years** | **AFT in % (95%-CI)** | **HR (95%-CI)** | **AFT in % (95%-CI)** | **HR (95%-CI)** |
| Cumulative bacteria-/protozoa score  (linear effect) | 250 | 4607 | -14.54 (-24.81; -4.27) | 1.29 (1.08; 1.55) | -13.28 (-22.54; -4.02) | 1.29 (1.08; 1.56) |
| Cumulative number of bacteria-/protozoa infections a person was seropositive for |  |  |  |  |  |  |
| *0 Infections (n=9)* | 14 | 314 | 3.18 (-28.67; 35.03) | 0.95 (0.54; 1.66) | -3.92 (-32.63; 24.79) | 1.08 (0.61; 1.9) |
| *1 Infections** (n=38)* | 87 | 1822 | 0 | 1 | 0 | 1 |
| *2 Infections (n=91)* | 127 | 2206 | -12.78 (-28.19; 2.63) | 1.25 (0.95; 1.65) | -16.19 (-30.16; -2.22) | 1.38 (1.04; 1.81) |
| *3 Infections (n=145)* | 22 | 265 | -40.66 (-67.16; -14.16) | 2.06 (1.29; 3.29) | -33.54 (-57.61; -9.46) | 1.94 (1.2; 3.12) |

*adjusted for age, sex, education years and a comorbidity index; **one infection as reference category

**Supplementary Table 7** Association between H. pylori IgG antibody titer quartiles and all-cause mortality (n=365)

|  |  |  | **Univariable analysis** | | **Multivariable analysis*** | |
| --- | --- | --- | --- | --- | --- | --- |
| ***H. pylori* quartile** | **Deaths** | **Follow-up years** | **AFT in % (95%-CI)** | **HR (95%-CI)** | **AFT in % (95%-CI)** | **HR (95%-CI)** |
| *H. pylori* quartiles (linear effect) | 250 | 4607 | -7.91 (-14.04; -1.78) | 1.15 (1.03; 1.28) | -4.24 (-9.85; 1.37) | 1.09 (0.97; 1.21) |
| Quartiles |  |  |  |  |  |  |
| *First (0.0 – 5.57)*** | 48 | 1307 | 0 | 1 | 0 | 1 |
| *Second (5.58 – 30.19)* | 69 | 1033 | -40.01 (-60.9; -19.12) | 2.04 (1.41; 2.95) | -28.48 (-47.44; -9.51) | 1.75 (1.21; 2.54) |
| *Third (30.2 – 102.28)* | 65 | 1126 | -29.29 (-50.36; -8.22) | 1.69 (1.16; 2.45) | -27.99 (-47.52; -8.46) | 1.73 (1.18; 2.55) |
| *Fourth (102.29– 150)* | 68 | 1141 | -31.88 (-52.75; -11.01) | 1.77 (1.22; 2.56) | -17.45 (-36.57; 1.67) | 1.41 (0.97; 2.05) |

*adjusted for age, sex, education years and a comorbidity index; **first quartile as reference (0 – 5.57)

**Supplementary Table 8** Association between B. burgdorferi s.l. IgG antibody titer quartiles and all-cause mortality (n=365)

|  |  |  | **Univariable analysis** | | **Multivariable analysis*** | |
| --- | --- | --- | --- | --- | --- | --- |
| ***B. burgdorferi s.l.* quartile** | **Deaths** | **Follow-up years** | **AFT in % (95%-CI)** | **HR (95%-CI)** | **AFT in % (95%-CI)** | **HR (95%-CI)** |
| *B. burgdorferi s.l.* quartiles (linear effect) | 250 | 4607 | -1.95 (-8.34; 4.44) | 1.03 (0.93; 1.16) | -4.19 (-10.01; 1.64) | 1.08 (0.97; 1.21) |
| Quartiles |  |  |  |  |  |  |
| *First (0.0 – 6.29)*** | 60 | 1166.36 | 0 | 1 | 0 | 1 |
| *Second (6.3 – 7.65)* | 62 | 1035.44 | -10.48 (-30.65; 9.69) | 1.2 (0.84; 1.72) | -16.19 (-34.6; 2.21) | 0.85 (0.71; 1.02) |
| *Third (7.66 – 12.61)* | 61 | 1302.48 | 6.05 (-14.2; 26.3) | 0.9 (0.63; 1.28) | -2.29 (-20.9; 16.3) | 0.98 (0.81; 1.18) |
| *Fourth (12.62– 200)* | 67 | 1102.69 | -12.0 (-31.8; 7.8) | 1.24 (0.87; 1.75) | -18.13 (-36.33; 0.07) | 0.83 (0.7; 1) |

*adjusted for age, sex, education years and a comorbidity index; **first quartile as reference (0 – 6.29)

**Supplementary Table 9** Association between the cumulative infection score and all-cause mortality stratified by age quartiles (n=365)

|  |  |  | **Univariable analysis** | | **Multivariable analysis*** | |
| --- | --- | --- | --- | --- | --- | --- |
| **Cumulative infection score** | **Deaths** | **Follow-up years** | **AFT in % (95%-CI)** | **HR (95%-CI)** | **AFT in % (95%-CI)** | **HR (95%-CI)** |
| Age quartiles (linear effect) |  |  |  |  |  |  |
| *First (65 – 69 years)* | 44 | 1471 | -17.28 (-33.43; -1.13) | 1.38 (1.03; 1.85) | -20.81 (-37.84; -3.78) | 1.52 (1.09; 2.13) |
| *Second (70 – 73 years)* | 67 | 1454 | -11.03 (-24.42; 2.36) | 1.23 (0.96; 1.58) | -10.51 (-24.08; 3.05) | 1.22 (0.94; 1.59) |
| *Third (74 – 76 years)* | 62 | 935 | -13.78 (-25.3; -2.56) | 1.35 (1.05; 1.73) | -13.7 (-25.17; -2.23) | 1.36 (1.05; 1.75) |
| *Fourth (77– 83 years)* | 77 | 747 | -10.94 (-21.21; -0.67) | 1.22 (1.01; 1.48) | -9.76 (-19.91; 0.39) | 1.21 (0.99; 1.47) |

*adjusted for age, sex, education years and a comorbidity index
