# Supplementary figures and images for: Seropositivity for pathogens associated with chronic infections is a risk factor for all-cause mortality in the elderly: findings from the Memory and Morbidity in Augsburg Elderly (MEMO) Study
Source: GeroScience. 2020 Jul 9;42(5):1365–76. doi: 10.1007/s11357-020-00216-x (PMC7525922; doi:10.1007/s11357-020-00216-x)

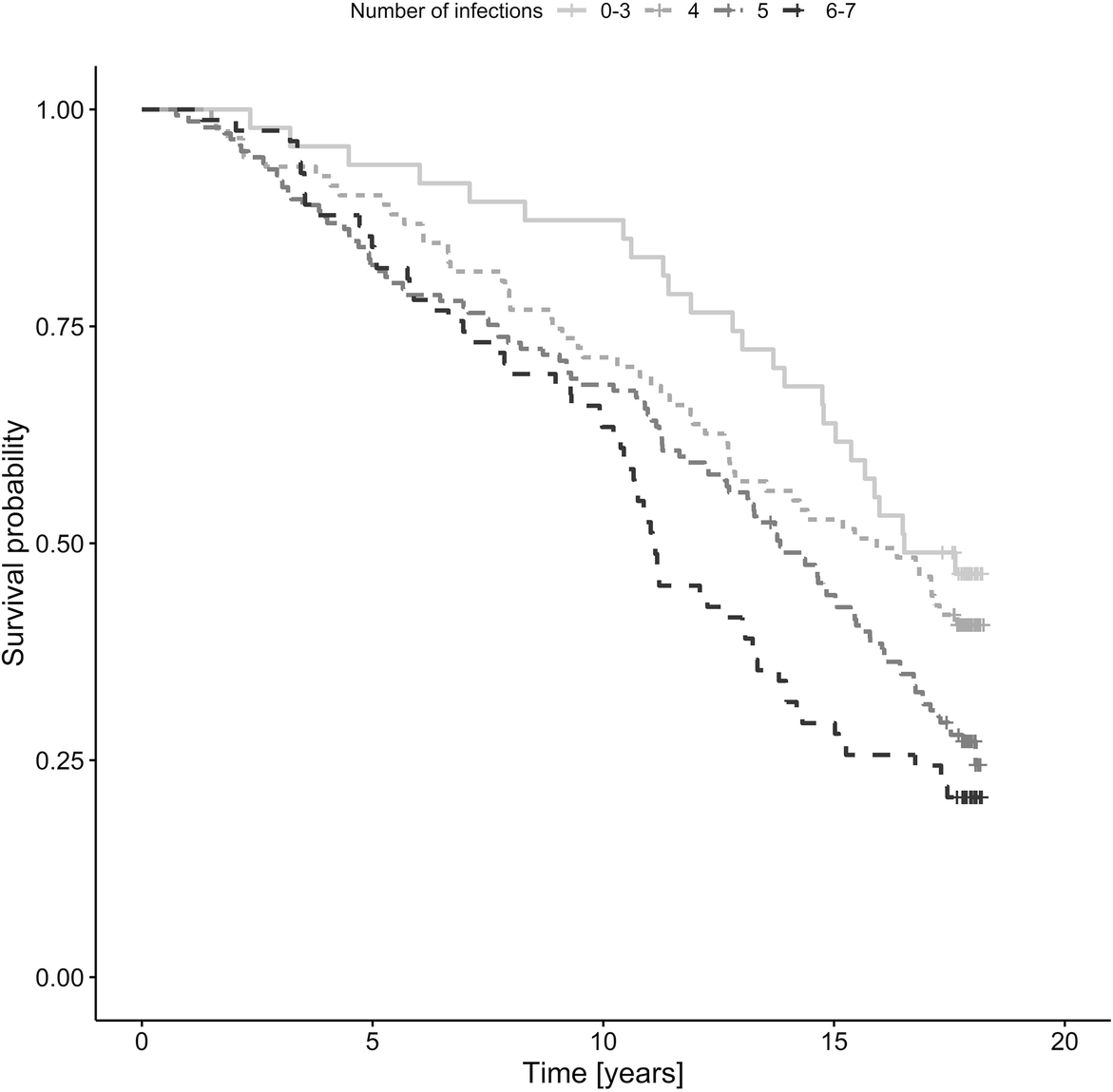

Supplement: Supplementary file 2 — Kaplan Meier survival curve by levels of the cumulative infection score, based on the sum of seropositive results (among CMV, HSV-1/2, HHV-6, EBV, H. pylori, T. gondii, and B. burgdorferi s. l.) (PNG 49.7 kb) [file 11357_2020_216_Fig4_ESM.png]

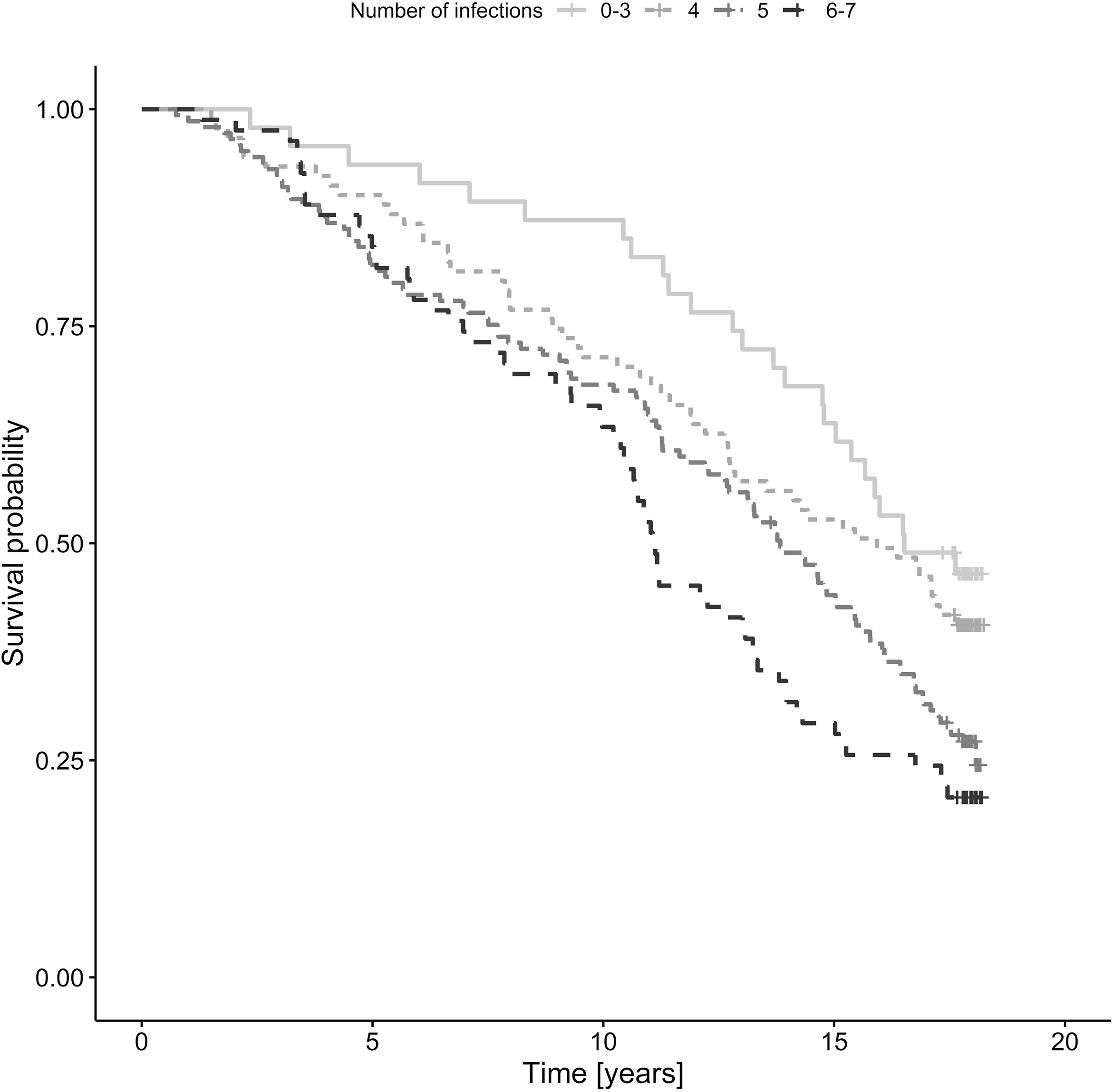

Supplement: Supplementary file 3 — High resolution image (TIF 134 kb) [file 11357_2020_216_MOESM2_ESM.tif]
